# Nutrition and Muscle Recovery

**DOI:** 10.3390/nu13020294

**Published:** 2021-01-20

**Authors:** Juan Mielgo-Ayuso, Diego Fernández-Lázaro

**Affiliations:** 1 Department of Health Sciences, Faculty of Health Sciences, University of Burgos, 09001 Burgos, Spain; 2 ImFINE Research Group, Department of Health and Human Performance, Faculty of Physical Activity and Sport Sciences-INEF, Universidad Politécnica de Madrid, 28040 Madrid, Spain; 3 Department of Cellular Biology, Histology and Pharmacology, Faculty of Health Sciences, University of Valladolid, Campus of Soria, 42003 Soria, Spain; diego.fernandez.lazaro@uva.es; 4 Neurobiology Research Group, Faculty of Medicine, University of Valladolid, 47002 Valladolid, Spain

Exercise-induced muscle damage (EIMD) is characterized by histopathological muscle tissue changes that originate skeletal muscle damage. The destruction of skeletal muscle fibers causes an inflammatory response that decreases the athlete’s physical work capacity and sports performance. Thus, muscle recovery becomes essential and has become a priority for elite athletes in different sports modalities. To achieve optimal muscle recovery, athletes often combine additional recovery strategies (biological, pharmacological, mechanical, and nutritional) in the hope of improving physiological responses and competitive performance. This extra preparation could contribute sensibly and legally to athletes to adequately complement their training to obtain better performance or try “shortcuts” to reach the sport’s elite in less time, with treatments and/or prohibited artificial methods that improve their ability to achieve more extraordinary physical performance. Among the strategies employed, the nutritional plan has a decisive influence on the stimulation of muscle recovery. However, it is necessary to optimize the consumption of adequate amounts of energy, nutrients, and liquids, establishing the correct frequency and associated with the temporality of training and competition.

Furthermore, there are occasions when all these nutritional indications are insufficient, and it becomes necessary to administer supplements to improve sports performance. Dietary supplements are intended to complete and enhance the athlete’s diet, optimize recovery during or after efforts, and increase the energy reserves needed to face strenuous competitions. For this reason, in this Special Issue, *Nutrition and Muscle Recovery*, we describe the most influential nutritional resources for promoting muscle anabolism. Studies on proteins, amino acids, carbohydrates, antioxidants, and dietary supplements have demonstrated their importance and effectiveness in muscle recovery. It is also essential to take into account the guidelines on quantity, time, and composition of each of the nutritional elements to maximize their effectiveness, taking into account the principle of sports specificity.

*Nutrients’* special edition has brought together various research manuscripts [1,2,3,4,5,6,7,8,9,10,11] and a systematic review [12]. This Special Issue, entitled *Nutrition and Muscle Recovery*, gathered 12 manuscripts [1,2,3,4,5,6,7,8,9,10,11,12]; one manuscript (8.3%) [9] was related to the analysis of the coach’s social skills influencing the athlete’s eating habits. In this way, the essential role of eating habits to attain sporting success is demonstrated. Trigueros et al. [9] included 1547 subjects, men and women in different team sports (soccer, basketball, volleyball, and handball), and 127 trainers. The main results showed that the psychological disorders derived from anxiety, stress, and depression directly influenced the patterns of unhealthy eating. Thus, these findings stimulate the implementation of a favorable social environment to develop nutritional strategies that encourage a diet that achieves optimal health for athletes to succeed in the sport. Trigueros et al. [9] showed that coaches’ respectful and understanding behavior with their athletes improves psychological and emotional well-being, self-esteem, and confidence. In this way, the athletes can face sports practice stressors and develop healthy eating habits that result in improvements in their sports performance.

Additionally, two research papers (16.6%) analyzed the nutritional composition of sports foods [3,6] that allows the generation of individualized diets according to the athlete’s sports performance and competitive performance. Martínez-Sanz et al. [3] generated a database that reports foods’ composition concerning the portion sizes usually consumed by athletes and/or commercial recommendation guidelines. Three hundred and twenty-two foods with a high interest in sports practice and 18 registered trademarks that provided nutritional data were analyzed. These foods were classified into seven categories: “sports drinks; sports gels; sports bars; sports confectionery; protein powders; protein bars; and liquid foods.” In this way, a tool was generated for the nutrition professionals that facilitates the athletes’ dietetic-nutritional planning before, during, or after the training and/or competition. Mielgo-Ayuso et al. [6] analyzed the associations between EIMD, cardiac stress (EICS), and the diets of marathon runners in the seven days prior to a competition. The results showed that semi-elite marathon athletes had higher levels of EIMD and EICS caused by the intake of meat in general, and butter and fatty meat in particular. In contrast, the consumption of fish, vegetables, and olive oil would exert a modulating effect on the EIMD and EICS [6]. With these results, appropriate nutrition education programs could be created for all sports professionals to achieve adequate health status to optimize sports performance.

In this Special Issue, two manuscripts (16.6%) [7,8] evaluated the comparative efficacy of whey proteins vs. vegetable-based proteins on EIMD. In this sense, Nieman et al. [7] evaluated comparatively, through a randomized trial, pea protein, serum protein, and placebo on muscle damage, inflammation, delayed onset of muscle pain (DOMS), and physical performance for five days after a 90-min high eccentric activity in a non-athletic, non-obese male population. These authors report that three doses of 0.3 g/kg per day of serum protein isolate during the five post-exercise days reduce muscle damage in the tested population. On the other hand, pea protein consumption had a minor effect on EIMD attenuation. Together, these data support using three doses of 0.3 g/kg per day of serum protein isolate during five days of recovery from intensive eccentric exercise to reduce serum levels of biomarkers of muscle damage in untrained men, with pea protein intake having an intermediate effect. Only the increase in muscle fiber size, muscle strength, and muscle recovery caused by pre-sleep serum protein intake was observed. However, both proteins used in the study (whey protein and pea protein) showed no significant decreases in DOMS and no increase in physical performance compared to placebo [7]. In this way, Saracino et al. [8] investigated to compare whey vs. plant-based (alternative protein sources) pre-sleep protein dietary supplementation on muscle recovery in 27 physically active middle-aged men. The results showed that the consumption of 1.08 ± 0.02 g/kg/day of the protein showed no effect on harmful eccentric exercise over 72 h. Pre-sleep protein intake, independent of protein source, did not mitigate muscle damage. For these reasons, Saracino et al. [8] state that adequate protein intake per day (1.2–1.6 g/kg or 1.4–2.0 g/kg) and a protein intake close to physical activity stimulate muscle recovery. In summary, these two studies [7,8] show that the development of lean mass, increased strength, and improved recovery are achieved through protein supplementation following the guidelines established in their research.

Carbohydrate (CHO) supplements may improve sports performance in certain physical activities of varying intensity and duration. In addition, during endurance exercise, CHO intake showed to delay neuromuscular fatigue and significantly improve physical work capacity, depending on the dose used and modulate EIMD biomarkers. Three studies (25%) [5,10,11] of this Special Issue, *Nutrition and Muscle Recovery*, investigated the effects of CHO ingestion on physical performance (repeated sprint efforts) [5], neuromuscular function [10], and EIMD markers [11]. A randomized, double-blind placebo-controlled crossover trial of 15 recreational athletes found that CHO ingestion immediately before and during short, maximal, and repeated sprint exercise does not influence performance and it does not increase the quality of training [5]. These findings question CHO’s potential ergogenic value for longer durations’ anaerobic performance than previously observed in other studies. McMahon et al. [5] provide some useful findings for prescribing CHO intakes for the athlete to perform practical performance-enhancing training. The CHO intake may not have been used to increase adenosine triphosphate (ATP) turnover, thus, improving anaerobic cycling performance compared to placebo. This type of CHO ingestion does not appear to provide any ergogenic benefits [5]. Two studies [10,11] from the same research group examined and compared the effects of high CHO intake (120 g/h) in terms of CHO intake recommendation (90 g/h) and regular CHO intake performed by ultra-endurance athletes (60 g/h) during a mountain marathon, on neuromuscular function, high intensity run capacity recovery, and EIMD in marathon elite runners. This research group [10,11] showed, for the first time, that the intake of CHO higher than currently recommended (up to 120 g/h) during an endurance test positively stimulates long-term neuromuscular recovery and modulates the decline in sports performance 24 h after the end of the mountain marathon and constitutes a suitable strategy for modulating EIMD [10,11]. These studies [10,11] provide new evidence on carbohydrate consumption in elite athletes with results that modify previous results that establish the intestinal absorption limit at 90 g/h through small bowel carriers. The 120 g/h of CHO does not produce adverse reactions in the gastrointestinal tract. To achieve this, athletes must be trained to perform the maximum possible individual intake of CHO (up to 120 g/h). The mixture of CHO in a ratio of glucose and fructose of 2:1 could be considered an optimal composition to ingest high CHO amounts (up to 120 g/h) [10,11].

Nutritional supplements were studied in four studies (33.2%) in this Special Issue [1,2,4,12]. Bazzucchi et al. [1] and Martin-Rincon et al. [4] have studied the effect of polyphenols on muscle recovery, EIMD, and muscle pain, with quercetin (Q) in monotherapy and quercetin plus Zynamite^®^ (mango leaf extract), respectively. Q is a flavonol-type polyphenol and Zynamite^®^ is a natural polyphenol; both have antioxidant and anti-inflammatory attributes that may stop EIMD and promote muscle recovery [1,4]. The supplementation with Q (1 gr/d) for 14 days following a double-blind crossover study design improve recovery from EIMD, the deterioration of neuromuscular function, and modulated the increase in muscular biomarkers such as creatine kinase (CK) and lactate-dehydrogenase (LDH) [1] in healthy men. Similarly, an only dose before 60 min the exercise (140 mg Zynamite^®^ plus 140 mg quercetin), followed by after 3 extra-doses every 8 h allowed modulates EIMD and stimulates the recovery of muscle performance [4]. Recovery of muscle strength and performance after intense exercise is enhanced by polyphenol supplementation, probably due to protection against oxidative damage that prevents post-exercise muscle soreness. These effects impact the redox process and factors acting at the central nervous system level by eliminating free radicals involved in nociception [1,4].

Athletes often turn to nutritional supplements such as proteins and amino acids to keep health and increase sports performance to the maximum. The proteins and amino acids represent the most consumed ergogenic aids. In this Special Issue, two manuscripts [2,12] described the effect of two amino acid supplementation strategies on sports performance. Fernandez-Landa et al. [2] created one of a small number of research studies of the additional effect level of amino acid mixing (creatine monohydrate (CrM) and β-hydroxy β-methylbutyrate (HMB)); also, they have identified whether their effects are synergistic in professional rowers. The main findings of these authors [2] are that the mixture of CrM plus HMB has a highly synergistic effect on anaerobic performance evaluated on 4 mmol and 8 mmol blood lactate concentration. The results of this research have an immediate practical application as the supplementation for 10 weeks HMB (3 g/day) plus CrM (0.04 g/kg/day) improves sports performance. Furthermore, this represents a novel way of evaluating supplementation strategies’ real effect from the research perspective.

There is only one review in this special volume. This is a systematic review and meta-analysis [12] to estimate the impacts of arginine (Arg) supplementation on sport performance. It additionally investigates the effective dose and timing of Arg. Eighteen randomized controlled clinical trials investigated Arg supplementation on the potential effect on aerobic and anaerobic performance tests. The meta-analysis results determined that the acute administration of Arg at 0.15 g/kg (10–11 g) 60–90 min prior to exercise stimulates substantial improvements in both aerobic and anaerobic exercise capacity. After chronic administration of Arg 1.5 to 2 g/day for 4 to 7 or more doses (10 to 12 g/day for 8 weeks), different relevant results showed improvements in athletic performance [12]. All studies in this systematic review and meta-analysis included Arg in monotherapy. This prevents the evaluation of possible additive effects with other ergo-nutritional supplements, such as the study developed by Fernandez-Landa et al. [2].

The diversity of articles published in the Special Issue *Nutrition and Muscle Recovery* highlights the role of diet, healthy behavior, nutritional strategies, and dietary supplements on muscle recovery to improve sports performance and/or reduce fatigue in sport.
